# New Species of Babinskaiidae (Insecta: Neuroptera) From the Mid‐Cretaceous of Myanmar and the Morphological Divergence of the Family Across the Cretaceous

**DOI:** 10.1002/ece3.73210

**Published:** 2026-03-09

**Authors:** Xiumei Lu, Yunlin Luo, De Zhuo, Xingyue Liu

**Affiliations:** ^1^ Institute of Ecological and Environmental Protection Shanghai Academy of Agricultural Sciences Shanghai China; ^2^ Bristol Palaeobiology Group, School of Earth Sciences University of Bristol Bristol UK; ^3^ Department of Entomology China Agricultural University Beijing China; ^4^ Beijing Xiachong Amber Museum Beijing China; ^5^ State Key Laboratory of Animal Biodiversity Conservation and Integrated Pest Management, Institute of Zoology Chinese Academy of Sciences Beijing China

**Keywords:** Babinskaiidae, disparity, diversity, Kachin amber, locality

## Abstract

Babinskaiidae is an extinct lacewing family, only known from the Cretaceous, of the superfamily Myrmeleontoidea, currently comprising 22 species in 16 genera. The family is primarily recorded from two Cretaceous deposits: the Early Cretaceous Crato Formation of Brazil and the mid‐Cretaceous Kachin amber of Myanmar. Shared morphology between the two localities points to a possible evolutionary or biogeographic link. Here, we describe two new species of Babinskaiidae from the mid‐Cretaceous Kachin amber: *Burmobabinskaia jiaxiaoae* sp. nov. and *Parababinskaia weijie* sp. nov. The combined presence of female gonapophysis 8 and gonocoxites 8 is documented for the first time in Babinskaiidae based on a new female specimen of *Burmobabinskaia*. Despite the occurrence of a shared genus between two deposits, the degree of morphological disparity within Cretaceous Babinskaiidae—and the character traits responsible for this variation—has not been quantified. To address this, we compared the morphological disparity of Babinskaiidae from the Crato Formation and the Kachin amber. Our results reveal pronounced morphological divergence between the two localities, both in overall size and morphospace orientation, with little overlap. A correlation between body length and the primary PCoA axis further indicates that size‐related traits may be key drivers of morphological variation within the family. The available niches in the Myanmar ecosystem may have accelerated adaptive evolution, propelling morphological divergence through ecological differentiation and lineage‐specific adaptations.

## Graphical abstract

Two new species of Babinskaiidae are reported from the mid‐Cretaceous of Myanmar and the morphological divergence of the family across the Cretaceous is quantified. The Babinskaiidae assemblages from the Lower Cretaceous Crato Formation and the Upper Cretaceous Kachin amber are separated by a pronounced morphological divergence in both overall size and morphospace orientation, with little overlap. A correlation between body length and the primary PCoA axis further indicates that size‐related traits may be key drivers of morphological variation within the family.

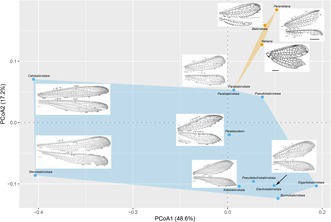

## 1 Introduction

Babinskaiidae is an extinct family within the superfamily Myrmeleontoidea. Together with the putative family ‘Cratosmylidae’, it represents a transitional lineage bridging the stem group Nymphidae and the crown myrmeleontoid families, including Nemopteridae, Palaeoleontidae, and Myrmeleontidae, based on previous phylogenetic analyses (Lu et al. 2021, 2022). Adults of Babinskaiidae are diagnosed by a unique combination of morphological characters, including long filiform antennae, a distally originating RP + MA vein, the presence of presectoral crossveins in both fore‐ and hind wings, absence of an oblique vein (the base of MP2) between the forewing MP and CuA, and reduction of the hind wing A2 and A3 veins in most species (Martins‐Neto and Vulcano 1989a; Lu et al. 2017, 2021, 2022).

Hitherto, 22 species in 16 genera have been described from three Cretaceous localities: the Lower Cretaceous Zaza Formation (Russia) and Crato Formation (Brazil), and the mid‐Cretaceous Kachin amber (Myanmar) (File S1; Martins‐Neto and Vulcano 1989a, 1989b; Ponomarenko 1992; Martins‐Neto 1997; Lu et al. 2017, 2021, 2022; Makarkin et al. 2017; Hu et al. 2018; Huang et al. 2019; Makarkin and Staniczek 2019; Ngô‐Muller et al. 2020; Jouault and Nel 2021; Jouault 2022; Pu et al. 2025). Among them, only a single species is known from the Zaza Formation (Ponomarenko 1992), while the remaining species are distributed between the Brazilian (6 species in 4 genera; Martins‐Neto 1997; Makarkin et al. 2017; Makarkin and Staniczek 2019; Jouault and Nel 2021; Ngô‐Muller et al. 2020) and Myanmar deposits (15 species in 12 genera; Lu et al. 2017, 2021, 2022; Makarkin et al. 2017; Hu et al. 2018; Huang et al. 2019; Makarkin and Staniczek 2019; Jouault 2022; Pu et al. 2025). These two localities have yielded a rich diversity of lacewings and exhibit overlapping taxa with similar specialized morphologies, such as leaf‐like antlions (Lu et al. 2019) and rare cratosmylids (Lu et al. 2022). Notably, one genus of Babinskaiidae is also shared between these two localities, that is, *Parababinskaia* Makarkin et al. 2017. However, the morphological disparity and evolutionary trajectory of Babinskaiidae across these two localities, as well as the potential ecological factors influencing such disparity, remain unquantified and poorly understood.

Here, we described two new species of Babinskaiidae: *Burmobabinskaia jiaxiaoae* sp. nov. and *Parababinskaia weijie* sp. nov., from the mid‐Cretaceous Kachin amber. These species exhibit previously undocumented characters of Babinskaiidae. *Burmobabinskaia* represents a monotypic genus defined by a distinctively narrowed hind wing, a morphology commonly found in Nemopteridae. This genus was previously established based on incomplete material (Lu et al. 2017); it is here redescribed from a well‐preserved specimen. The latter is characterized by the presence of six presectoral crossveins in the hind wing, compared to the typical 3–4 preserved in most babinskaiids. We also presented a comparative analysis of morphological disparity of Babinskaiidae from the Crato Formation and Kachin amber, based on an updated dataset of discrete morphological characters (Lu et al. 2021, 2022), and our quantitative analysis sheds new light on the morphological variation, evolutionary patterns, and ecological differentiation within this distinctive Cretaceous lacewing family.

## 2 Material and Methods

### 2.1 Taxonomy

The amber samples described are from the Hukwang Valley in Tanai Township, Myikyina District of Kachin State, Myanmar (Kania et al. 2015). The age of this deposit has been investigated and dated to be 98.8 ± 0.6 million years by U–Pb dating of zircons from the volcanoclastic matrix of the amber (Shi et al. 2012).

The holotype of *Burmobabinskaia jiaxiaoae* sp. nov. is deposited in the Century Amber Museum (CAM), Shenzhen, and the holotype of *Parababinskaia weijie* sp. nov. is deposited in the Xiachong Amber Museum (XAM), Beijing, China (Table S1).

Photographs and drawings were taken or made using a Zeiss SteREO Discovery V12 microscope system connected with a Nikon D850 camera system. The figures were prepared with Adobe Photoshop CC 2019. Terminology of wing venation generally follows Aspöck et al. (1980) and Martins‐Neto (2000). Terminology of genitalia follows Aspöck and Aspöck (2008).

Abbreviations used for wing veins and cells are as follows: A, anal vein; C, costa; Cu, cubitus; CuA, cubitus anterior; MP, media posterior; R, radius; RA, radius anterior; RP, radius posterior; ScA, subcostal anterior; ScP, subcostal posterior; ps, presectoral crossvein.

The published work and the taxonomic acts it contains have been registered with ZooBank: urn:lsid:zoobank.org:pub:F6153587‐4493‐4363‐A77F‐7CB5B46AAD57.

### 2.2 Morphospace Divergence Between Two Localities

The Kachin amber of Myanmar and the Crato Formation of Brazil are separately from Upper and Lower Cretaceous deposits. The Kachin amber dates to the earliest Cenomanian, and the Crato Formation is a major sedimentary unit of the Upper Aptian, located in the Araripe Basin of northeastern Brazil.

We updated the morphological matrix of Lu et al. (2022) mainly by adding *Pseudoelectrobabinskaia* Pu et al. 2025, the two new species described here and kept the analyzed data only for Babinskaiidae (File S2). Three genera, that is, *Baisonelia* Ponomarenko, 1992, *Pseudoneliana* Huang et al. 2019, *Microbabinsksia* Pu et al. 2025 are not included due to the incomplete preservations. Therefore, the qualitative characters are mainly from diagnostic characters such as wing venations, genitalia and legs. The percentage of the missing data is 9.4%. The morphological character matrix was used to calculate a pairwise maximum observable rescaled distances (MORD) matrix with the function “calculate_morphological_distances” of the package Claddis v0.6.3 (Lloyd 2016) and then performed the principal coordinates analysis (PCoA), all using the function ‘cmdscale’ of the package stats v4.2.2 (R Core Team 2022). The localities of each genus are calculated (File S3). The plot was performed with ‘ggplot’ of the package ggplot2 (Wickham 2016). The species respectively from the Crato Formation and the Kachin amber were separately highlighted with convex hulls in the morphospace.

### 2.3 Correlation of Primary PCoA Axes and Body/Forewing Length

General body length and forewing length data for all known babinskaiids from the two localities were collected (File S4). We adopted the assessments for nearly completely preserved specimens. For species represented by multiple specimens, the mean value of each measurement was calculated.

The bar of genera and its length is plotted with ‘ggplot’ of the package ggplot2 (Wickham 2016). We calculated the correlation between body/forewing length and the first two PCoA axes (File S4) with the ‘lm’ function in the stats package. All statistical computing was performed with R v4.2.2 (R Core Team 2022) within the RStudio environment.

## 3 Results

### 3.1 Systematic Paleontology

**Order Neuroptera Linnaeus, 1758.**

**Superfamily Myrmeleontoidea Latreille, 1802**.

**Family Babinskaiidae Martins‐Neto and Vulcano** 1989a.

Type genus: *Babinskaia* Martins‐Neto and Vulcano 1989a.

***Genus Burmobabinskaia* Lu et al.** 2017.

**FIGURE 1:**
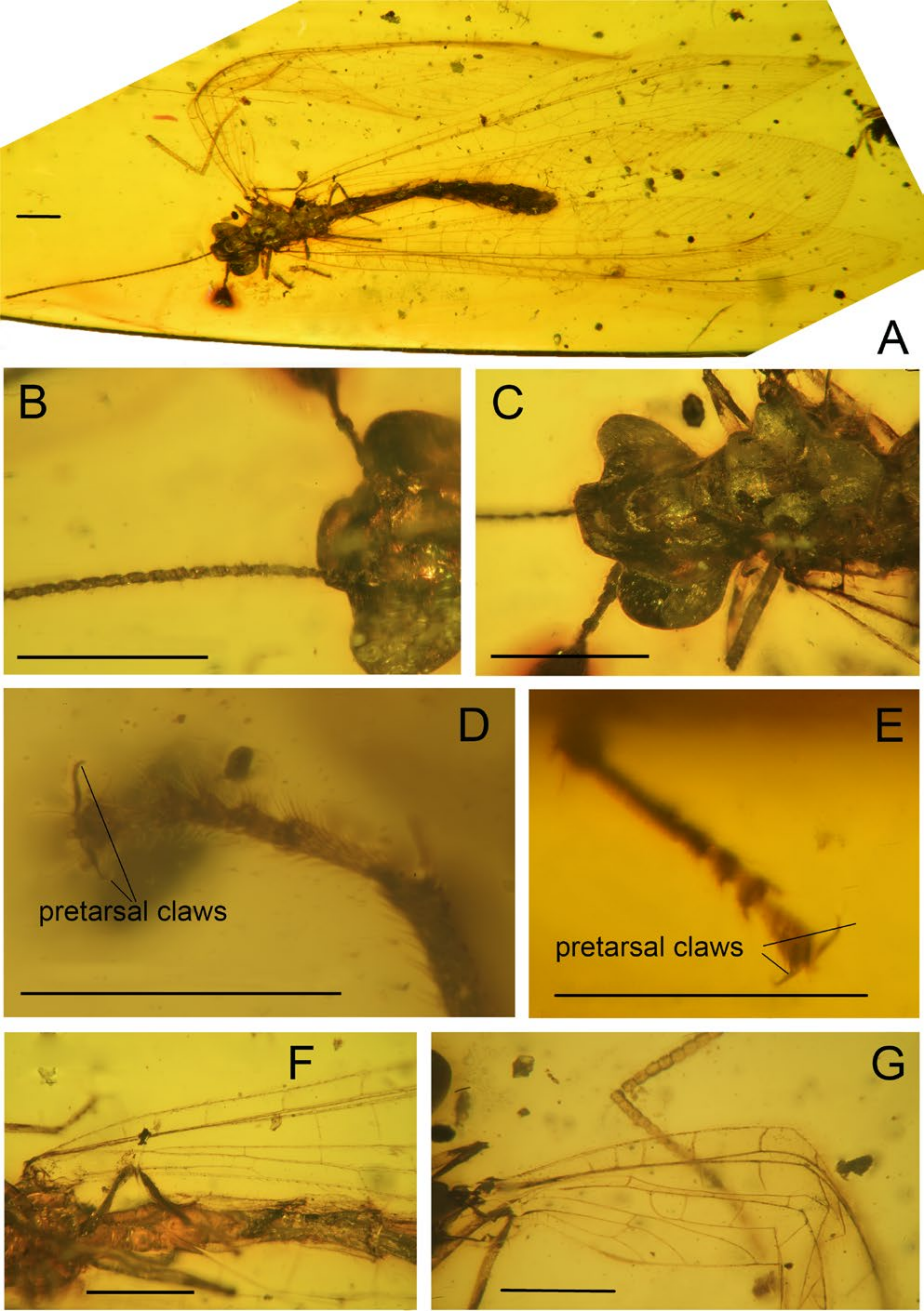
*Burmobabinskaia jiaxiaoae* sp. nov., holotype, CAM BA‐BAB‐25001. (A) habitus photograph, dorsal view; (B) photograph of antenna; (C) photograph of head and thorax; (D) photograph of pretarsal claw of the left hind leg; (E) photograph of pretarsal claw of the right mid‐leg; (F) photograph of base of right hind wing; (G) photograph of base of right forewing. Scale bar = 1.0 mm (A–C, F, G); 0.5 mm (D, E).

**FIGURE 2:**
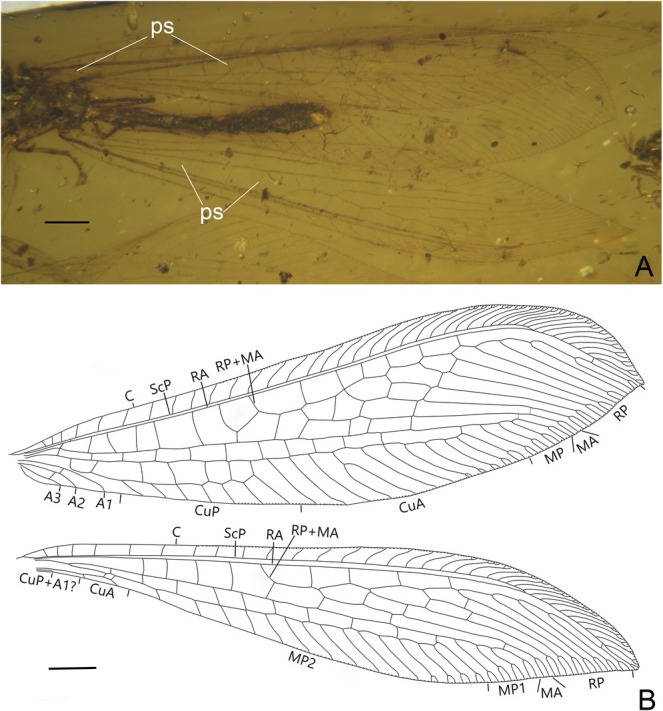
*Burmobabinskaia jiaxiaoae* sp. nov., holotype, CAM BA‐BAB‐25001. (A) photographs of wings; (B) drawings of wings. Scale bar = 0.5 mm.

**FIGURE 3:**
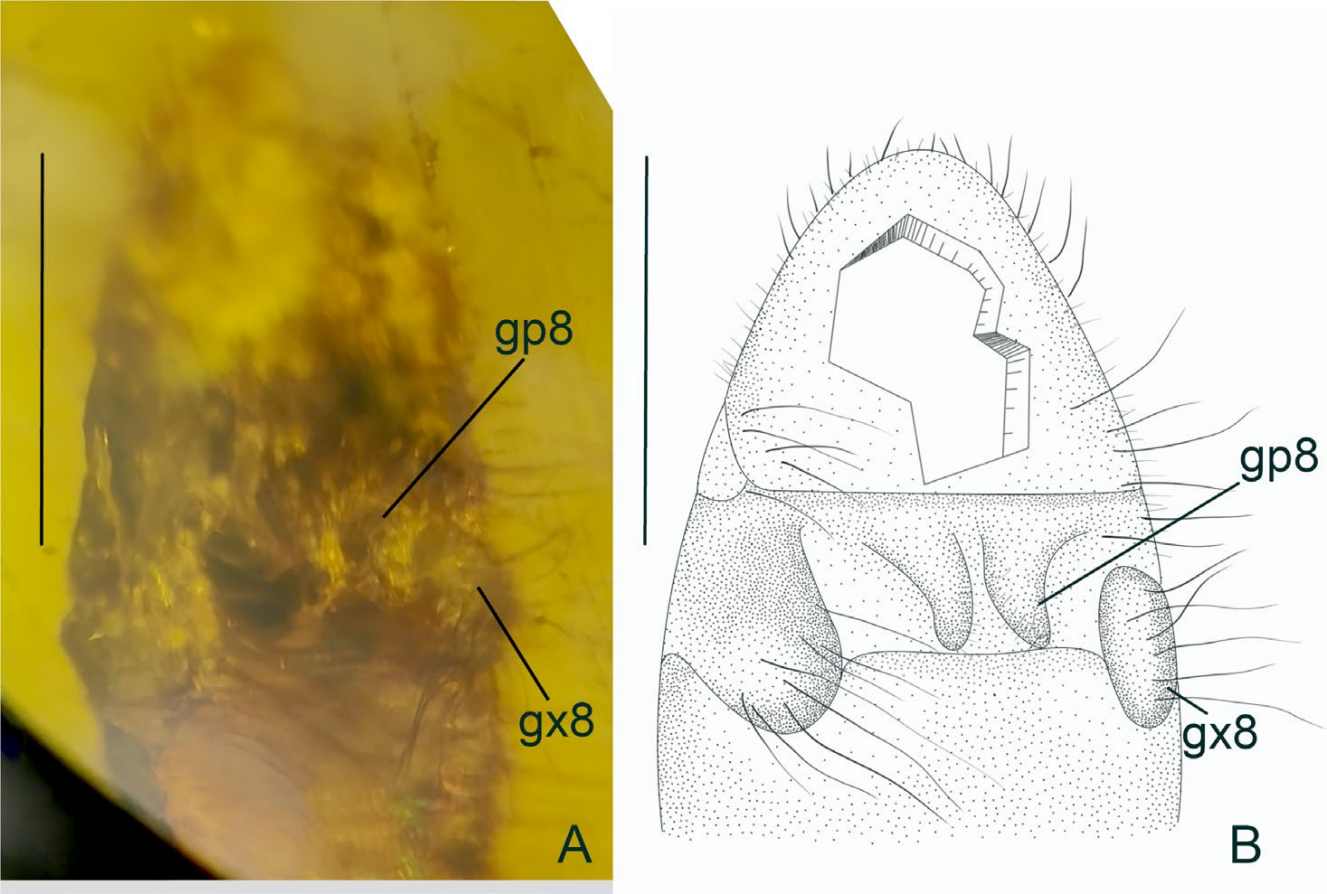
*Burmobabinskaia jiaxiaoae* sp. nov., holotype, CAM BA‐BAB‐25001. (A) photographs of female genitalia, ventral view; (B) drawing of female genitalia, ventral view. gp, gonapophysis; gx, gonocoxite. Scale bar = 1.0 mm.

Type Species: *Burmobabinskaia tenuis* Lu et al. 2017 (Figures 1, 2, 3). Described species: *Burmobabinskaia tenuis* Lu et al. 2017; *Burmobabinskaia jiaxiaoae* sp. nov.

**Revised diagnosis**. Adult medium‐sized, with body length ca. 8.0–10.2 mm. Forewing narrowly elongate, transparent and immaculate; at least five presectoral crossveins present; RP + MA originating proximal of the termination of CuP; RP with nine branches, most of them have a marginal fork; seven crossveins present between RA and RP; MP pectinately branched, distally with four branches, each bears a marginal fork; CuA pectinately branched, mostly simple except the distal‐most ones; CuP stem zig‐zagged distally, with seven simple branches; A1, A2 and A3 present and simple; two incomplete gradate series of crossveins present. Hind wing base strongly narrowed; three or four presectoral crossveins present; RP + MA originating distinctly distad termination of CuA; RP with seven branches, each bears a marginal fork; five crossveins present between RA and RP; MP1 pectinately branched distally, with four branches, each bears a marginal fork; MP2 long, pectinately branched, with most branches simple, except the distal‐most ones; CuA short, with only one marginal fork; CuP strongly reduced to a very short veinlet; A1 reduced to a short veinlet or totally absent; A2 and A3 absent. Male genitalia: Sternum 8 distinctly prominent ventrad; tergum 9 strongly extending and broadened ventrad; sternum 9 invisible, small and covered by tergum 9; ectoprocts paired, ovoid, with large callus cerci; a slender sclerite (putative part of gonocoxites and gonostyli 9) extending beyond ectoprocts. Female genitalia: Sternum 6 without projection; paired gonapophysis 8 and gonocoxites 8 present; gonocoxites 8 elliptical; gonapophysis 8 digitiform and slightly curved.

***Burmobabinskaia jiaxiaoae* sp. nov**.

LSID: urn:lsid:zoobank.org:act:5E6A5CD7‐3E54‐4518‐895F‐CCF131D1A007 (Figures 1, 2, 3).

**Diagnosis**. Wing base not narrowed; seven presectoral crossveins present in forewing and four presectoral crossveins present in hind wing; A1 reduced in hind wing.

**Description**. Female. Body length 8.0 mm; head 0.8 mm long and 1.4 mm wide; antenna length 6.3 mm; left forewing 13.4 mm long and 3.5 mm wide; right hind wing 13.0 mm long and 2.4 mm wide; abdomen length 5.2 mm.

Head orthognathous, almost triangular. Compound eyes large, semi‐globular; ocelli absent. Antenna filiform, with dense short setae; scape longer than pedicel; flagellomere narrower and almost as long as scape.

Prothorax slightly narrower but longer than head, laterally with long hair; meso‐ and metathorax robust. Wings slightly elongated, transparent and immaculate.

Forewing ca. 3–4 times as long as wide; multiple trichosors (up to seven) between veins along costal and posterior margins. Costal space about eight times as wide as subcostal space, but slightly narrower than radial space, with 21 simple veinlets on proximal 2/3 and 20 marginally forked, more inclined veinlets on distal 1/3; only one subcostal crossvein (1scp‐r) present near the wing base. Seven presectoral crossveins present, an oblique veinlet present connecting distal‐most presectoral crossvein and stem of RP + MA. RP + MA originated from R at proximal 1/3 of wing. Origin of RP + MA slightly proximal of termination of CuP. RP pectinately branched, with nine branches, most of which bearing a marginal fork. Two incomplete gradate series crossveins present. MA diverging from RP approximad diverging of RP + MA from RA, with one marginal fork. MP long and nearly straight, with 12 crossveins present between MP and CuA; no oblique vein (i.e., stem of MP2) present. CuA and CuP diverging near wing base. CuA distally feebly zig‐zagged along its stem, pectinately branched with 11 branches, half of which are simple, the fourth deeply forked, each of the distal‐most five branches with a small marginal fork. CuP pectinately branched, with seven simple branches. Six cua‐cup crossveins present. A1 simple, approximate basal CuP, without cup‐a1 crossvein; A2 and A3 short and simple, connected with a short crossvein.

Hind wing narrower than forewing, proximally distinctly narrow, increasingly broadened distad, with a sharp and slightly posteriorly curved wing apex. Trichosors as in forewing. Costal space about three times as wide as subcostal space, with 17 simple veinlets on proximal 2/3 and 17 marginally forked, more inclined veinlets on distal 1/3. Subcostal crossvein absent. Four presectoral crossveins present. RP pectinately branched with seven branches, each bearing a marginal fork. MA with a marginal fork; MP originated near R, MP1 and MP2 diverging near wing base; MP1 straight and long, pectinately branched at its distal 1/5, with four branches, each bearing a marginal fork; MP2 pectinately branched, with 15 branches, most of which are simple, except the posterior‐most four branches each with a marginal fork. Ten mp1‐mp2 crossveins present. CuA short, with two simple branches. CuP strongly reduced as a short veinlet near wing base.

Legs slender, with dense short setae; no specialized setae present. Tarsus 5‐segmented; tarsomere 1 slenderly elongated, tarsomeres 2–4 distinctly prominent on distal‐lateral corner; tarsomere 5 ovid; pretarsal claws equal in length and shape, slightly shorter than tarsomere 5, without additional tooth.

Abdomen slenderly elongated, with dense long setae; Female genitalia: Sternum 6 without projection; paired gonapophysis 8 and gonocoxites 8 present; gonocoxites 8 elliptical; gonapophysis 8 digitiform and slightly curved.

**Type material**. Holotype: CAM BA‐BAB‐25001: amber piece preserving a complete female of *Burmobabinskaia jiaxiaoae* sp. nov.; it is polished in the form of a flattened pentagon cabochon, clear and transparent, with length × width about 41.0 × 11.0 mm, height about 6.0 mm.

**Etymology**. The new species is dedicated to Mrs. Xiao Jia, who kindly offered the specimen (the holotype) of this new species for present study.

**Remarks**. The new species is assigned to *Burmobabinskaia* by the small body size, the presence of an oblique veinlet connecting the distal‐most presectoral crossvein and stem of RP + MA in the forewing, and the CuP with only one fork in the hind wing. However, it differs from *B. tenuis* by the presence of seven presectoral crossveins in the forewing and four presectoral crossveins in the hind wing, which are respectively five and three in *B. tenuis* . The CuP and A1 in the hind wing are separated in *B. tenuis* but the A1 is completely lost in the new species. In addition, the wing base of the new species is not as narrow as in *B. tenuis* .

***Genus Parababinskaia*** Makarkin et al. 2017.

**FIGURE 4:**
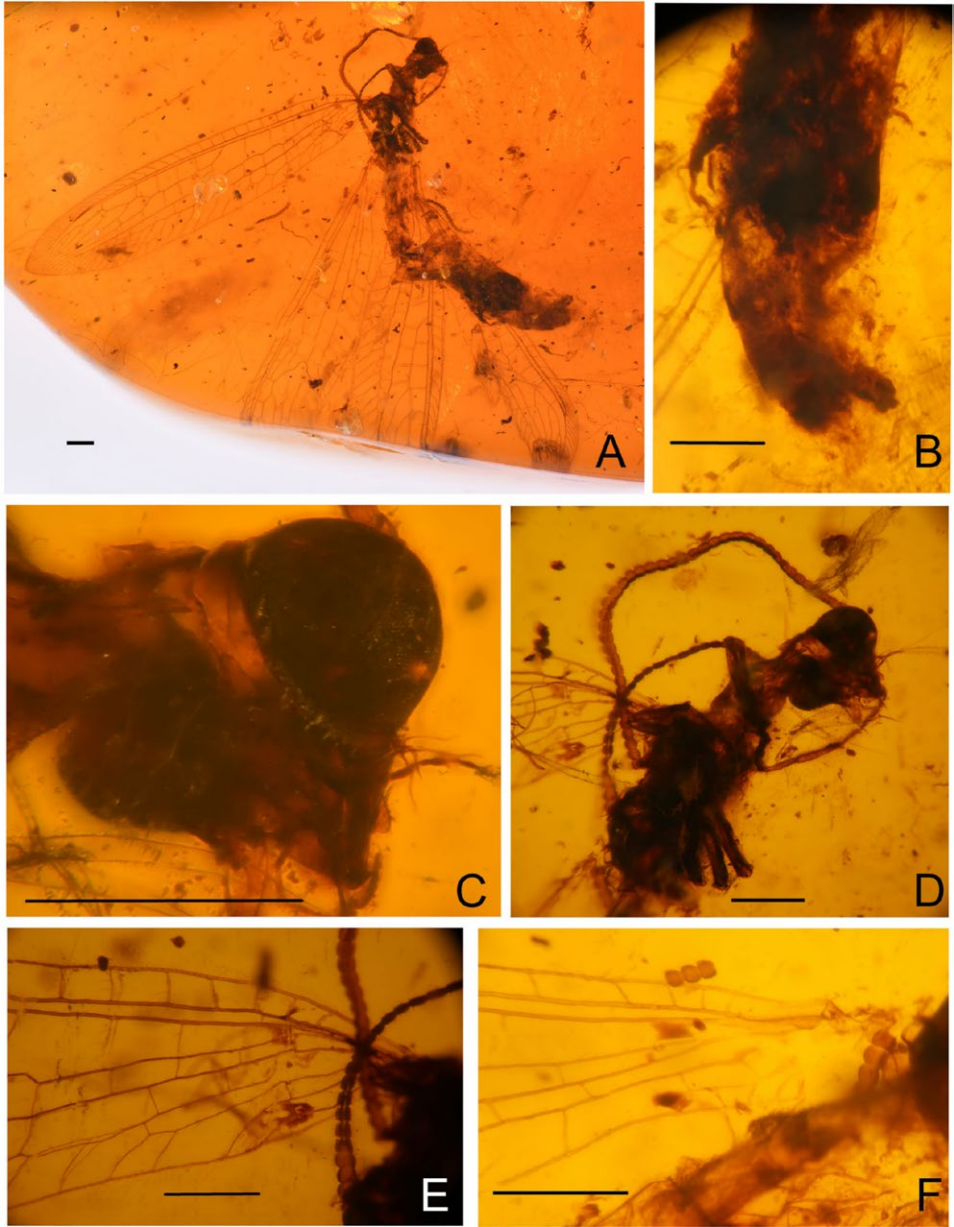
*Parababinskaia weijie* sp. nov., holotype, BXAM‐BAB‐25004. (A) habitus photograph, lateral view; (B) photograph of abdomen; (C) photograph of head; (D) photograph of antenna; (E) photograph of base of left forewing; (F) photograph of base of left hind wing. Scale bar = 1.0 mm.

**FIGURE 5:**
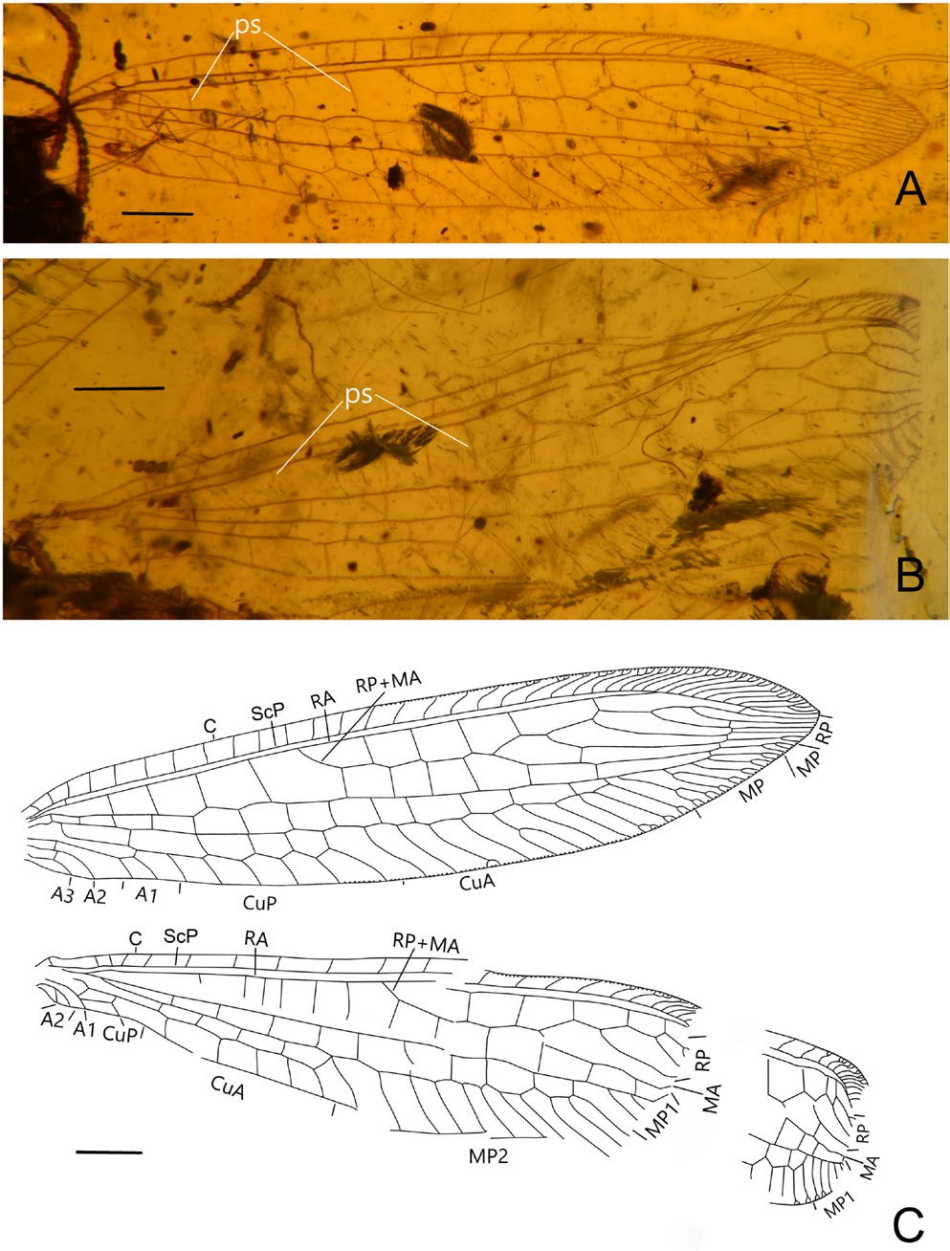
*Parababinskaia weijie* sp. nov., holotype, BXAM‐BAB‐25004. (A) photograph of forewing; (B) photograph of hind wing; (C) drawings of fore‐ and hind wings. Scale bar = 1.0 mm.

Type species: *Parababinskaia elegans* Makarkin et al. 2017 (Figures 4 and 5). Described species: *Parababinskaia elegans* Makarkin et al. 2017; *Parababinskaia makarkini* Hu et al. 2018; *Parababinskaia douteaui* Ngô‐Muller et al. 2020; *Parababinskaia weijie* sp. nov.

**Revised diagnosis**. Adult medium‐sized, with body length ca. 7.0–16.0 mm. Forewing narrowly elongate, transparent and immaculate; four to six presectoral crossveins present; RP + MA originating proximal of the termination of CuP; RP with five or six branches, most of which are simple; six crossveins present between RA and RP; MP pectinately branched ca. at distal 1/5; CuA pectinately branched; A1 bifurcated; A2 and A3 present, not fused with each other; one short outer gradate series of crossveins present. Hind wing slightly narrower than forewing, with three, four or six presectoral crossveins; RP + MA originating almost at same level with termination of CuA; RP with five branches; five to eight ra‐rp crossveins present; MP1 pectinately branched approx. at distal 1/5; MP2 pectinately branched nearly at its midpoint; CuP and A1 proximally fused in most species; A2 present. Female abdominal segment 6 without projections on sternum.

***Parababinskaia weijie* sp. nov**.

LSID: urn:lsid:zoobank.org:act:298BA776‐DAAA‐467E‐A930‐C639B547142A (Figures 4 and 5).

**Diagnosis**. Most forewing CuA branches simple; forewing CuP and A1 basally unfused; hind wing with six presectoral crossveins.

**Description**. Gender unknown. Body length 16.0 mm; head 1.6 mm long, 1.4 mm wide; partly preserved antenna length 11.0 mm; forewing 14.0 mm long, 3.0 mm wide; partly preserved hind wing 3.0 mm long, 6.0 mm wide; abdomen length 7.0 mm.

Head orthognathous, triangular. Wing venations covered with long hair. Compound eyes big, prominent, semi‐globular; ocellus absent; diameter of compound eye slightly wider than head width. Antenna filiform, with dense short setae; flagellum with at least 42 segments, proximal flagellomere slightly longer than width, distal flagellomere slightly wider than length. Labium palp three segments.

Prothorax almost as long as head; mesothorax wider than head; metanotum short. Wings narrowly elongated, transparent, and immaculate.

Forewing ca. 4–5 times as long as wide; multiple trichosors (up to six) between veins along costal and posterior margins. Costal space ca. four times as wide as subcostal space, but much narrower than radial space, with 18 simple veinlets on proximal 2/3 and 17 marginally forked, more inclined veinlets on distal 1/3; one subcostal crossvein (1scp‐r) present near the wing base. Four presectoral crossveins present. Origin of RP + MA proximal of termination of CuP. MA diverging from RP distad separating point of RA and RP + MA; RP with five branches, and only anterior‐most one bearing a small marginal fork. Five crossveins present in radial space. MA with three marginally pectinate branches. MP long and straight, pectinately branched about at its distal 1/5, and the two anterior‐most branches deeply bifurcated, the others with a small marginal fork. A short outer gradates series crossveins present. Fourteenth mp‐cua crossveins present. CuA and CuP diverging near wing base. CuA pectinately branched, with 10 branches, most of which are simple, the fifth deeply forked, each of the distal‐most three branches with a small marginal fork. CuP distally zig‐zagged along its stem, pectinately branched, with six simple branches. Five cua‐cup crossveins present. A1 distally bifurcated. No basal cup‐a crossvein present. A2 and A3 short and simple, not fused with each other.

Hind wing slightly narrower than forewing. Trichosors as in forewing. Costal space nearly two times as wide as subcostal space, with 15 simple veinlets on proximal 3/4 while remaining preserved marginally forked. Subcostal crossvein absent. Six presectoral crossveins present. RP + MA almost originating nearly at the same level of termination of CuA; RP with at least four RP branches. Five crossveins present in radial space. MP1 and MP2 diverging near wing base; MP1 straight and long, pectinately branched at its distal 1/5, with at least five branches, most of which bearing a marginal fork; MP2 slightly zig‐zagged distally, with 11 branches; most completely preserved branches are simple. Ten mp1‐mp2 crossveins present. CuA short, with five simple branches, distally zig‐zagged. CuP and A1 proximally fused. A1 simple, A2 present, short and bifurcated. Jugal lobe present.

**Type material**. Holotype: BXAM‐BAB‐25004: amber piece preserving an almost complete adult of *Parababinskaia weijie* sp. nov. (wings partly not preserved); it is polished in the form of a flattened pentagon cabochon, clear and transparent, with length×width about 37.0 × 29.0 mm, height about 19.0 mm.

**Etymology**. The new species is dedicated to Mr. Wei Zhang and Mrs. Hongjie Li. The mother of them contributed to the collection of this specimen for Mr. De Zhuo, who kindly offered this specimen for the present study.

**Remarks**. The new species is placed within *Parababinskaia* by the similar wing venation such as the similar number of presectoral crossveins and the forked A1 in the forewing. However, it differs from all other three species of *Parababinskaia* by the presence of six presectoral crossveins in the hind wings, whereas other species typically have only three or four presectoral crossveins. Additionally, it can be distinguished from *P. makarkini* by the forewing CuA branches, which are mostly simple in the new species but marginally forked in *P. makarkini*. It is also distinguishable from *P. douteaui* by having more costal veinlets with marginal forks and less presectoral crossveins in the forewing. Furthermore, the new species differs from both *P. makarkini* and *P. douteaui* in the configuration of forewing CuP and A1, which are basally unfused in the new species but fused or connected with a short crossvein in the latter two species. This character remains unknown in *P. elegans* .

### 3.2 Morphospace Divergence Between the Crato Formation and Kachin Amber

**FIGURE 6:**
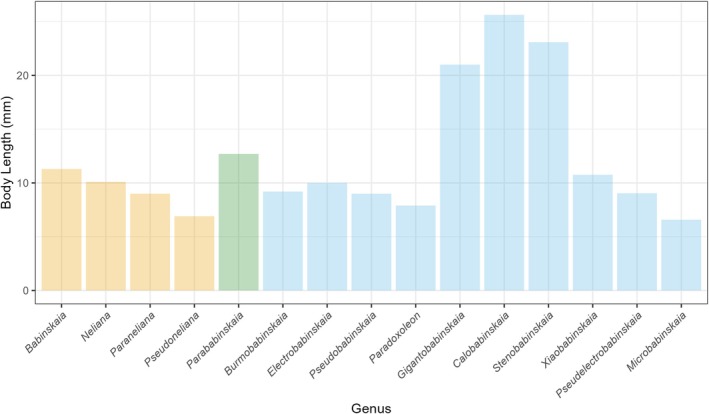
Bar plot of body length of genera of Babinskaiidae from the two localities, highlighting several genera of Kachin amber possess distinctly larger body‐size than the others. Yellow bars represent genera only from the Lower Cretaceous Crato Formation of Brazil; Blue bars represent genera only from the mid‐Cretaceous Kachin amber of Myanmar; Green bar represents genera shared by both localities. Body lengths of species are given in File S4.

**FIGURE 7:**
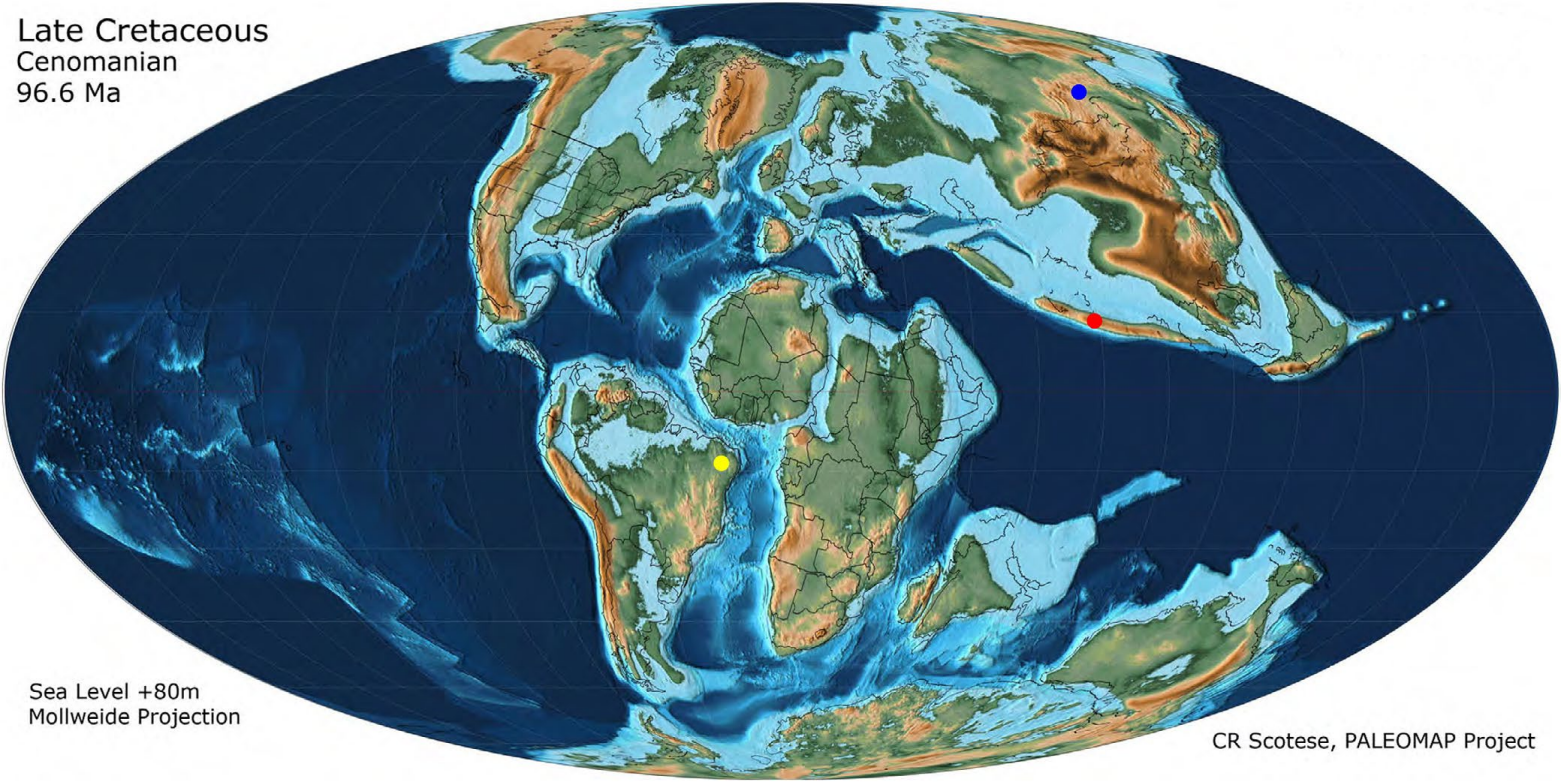
Paleogeographic map of the Upper Cretaceous (Cenomanian, 96.6 Ma) showing approximating localities of Babinskaiidae (the map is cited from Scotese 2014). Blue point represents the Zaza Formation, Baissa, Siberia, Russia; yellow point represents the Crato Formation, Nova Olinda, Ceara, Brazil; red point represents Tanai, Myitkyina, Kachin, Myanmar.

**FIGURE 8:**
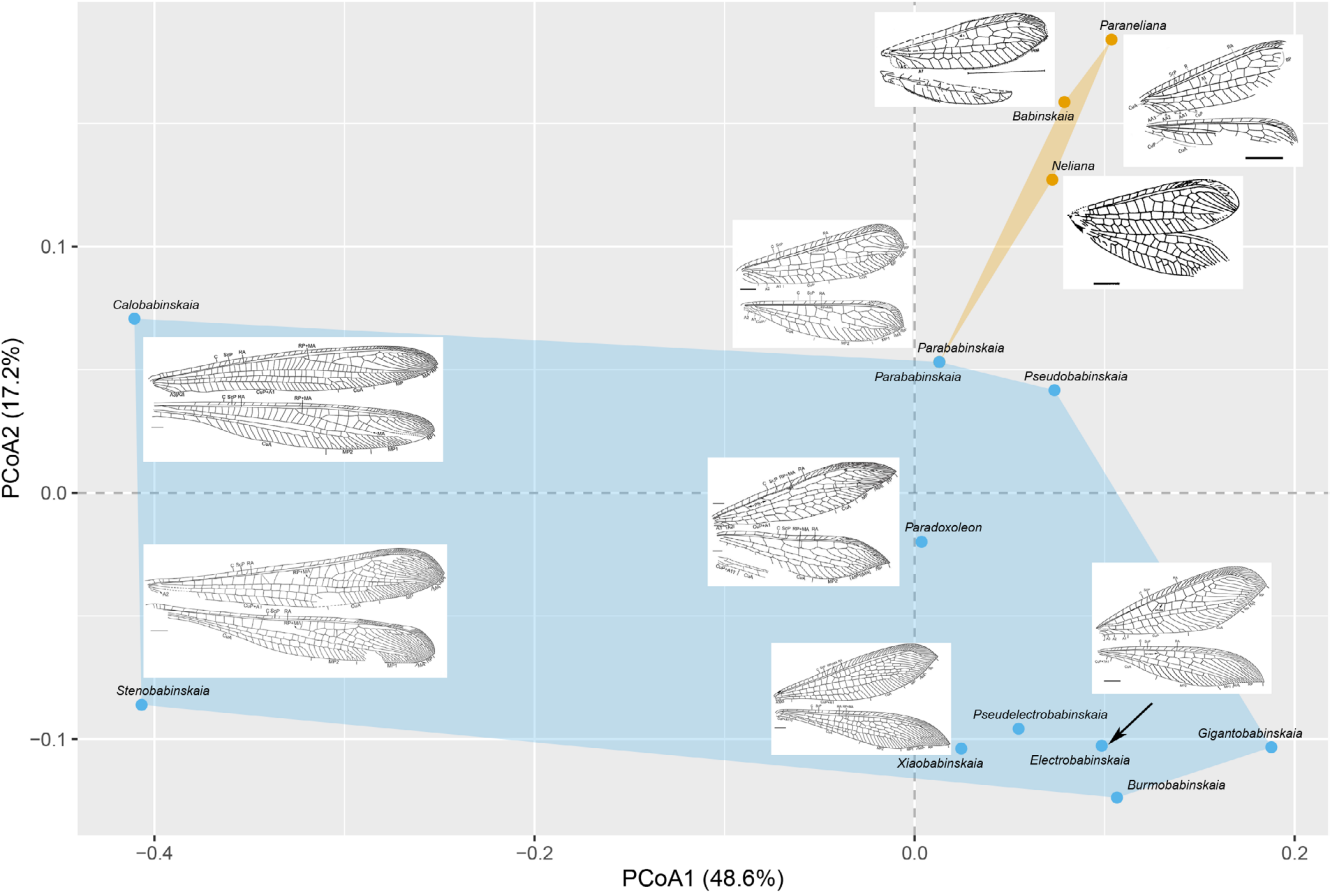
Morphospace of Babinskaiidae highlighting the morphological variation of genera from two different Cretaceous localities. Blue points represent morphospace of Babinskaiidae from the mid‐Cretaceous Kachin amber of Myanmar, while yellow points represent that from the Lower Cretaceous Crato Formation of Brazil. Wing images are cited from previous published works including those describing *Babinskaia pulchra* Martins‐Neto and Vulcano, 1989a (cited from Martins‐Neto 2000), *Neliana maculata* Martins‐Neto and Vulcano, 1989a (cited from Martins‐Neto 2000), *Electrobabinskaia burmana* Lu et al. 2017 (cited from Lu et al. 2017), *Parababinskaia makarkini* Hu et al. 2018 (cited from Hu et al. 2018), *Xiaobabinskaia lepidotricha* Lu et al. 2021 (cited from Lu et al. 2021), *Paraneliana sennlaubi* Jouault and Nel, 2021 (cited from Jouault and Nel 2021), *Calobabinskaia xiai* Lu et al. 2021 (cited from Lu et al. 2021), *Stenobabinskaia punctata* Lu et al. 2021 (cited from Lu et al. 2021), *Paradoxoleon chenruii* Lu et al. 2022 (cited from Lu et al. 2022).

The bar plot shows the body length of genera from the two localities (Figure 6). The three known Babinskaiidae localities are highlighted on a paleogeographic map of the Upper Cretaceous (Cenomanian, 96.6 Ma) (Figure 7). For morphospace, the first two principal components, PCoA1 and PCoA2, collectively explain 65.8% of the total morphological variation, effectively distinguishing groups of the two localities. PC1 contributes 48.6%, while PC2 contributes 17.2% to the overall variation (Figure 8). The results show that Babinskaiidae from the Crato Formation of Brazil and Kachin amber have distinctly different occupations in both size and ordination in the morphospace. The occupation of Babinskaiidae from Kachin amber is much larger than that from Brazil (Figure 8).

### 3.3 Correlation of PCoA Axis and Body Length

The correlation test showed that body length is statistically significant, moderately correlated to PCoA1 (*R* ^2^: 0.5227; *p*‐value: 0.005234) but not PCoA2 (*R* ^2^: 0.0115; *p*‐value: 0.7273), while forewing length showed only a weak and marginally significant relationship with PCoA1 (*R* ^2^: 0.3501; *p*‐value: 0.03316) and no significant association with PCoA2 (*R* ^2^: 0.1016; *p*‐value: 0.2884).

## 4 Discussion

### 4.1 Novel Morphological Characters of New Species

The two new species, *Burmobabinskaia jiaxiaoae* sp. nov. and *Parababinskaia weijie* sp. nov. undoubtedly belong to Babinskaiidae based on the presence of a combination of autapomorphies of this family, including the RP + MA diverging from a position distinctly distal to the wing base in both fore‐ and hind wings, and the presence of a single forewing MP (Lu et al. 2021, 2022).

The discovery of the two new species expanded our knowledge and understanding of this extinct family. *Burmobabinskaia* is previously represented by a monotypic male species, *B. tenuis* , described based on an incomplete specimen. *B. tenuis* possesses a distinctly narrowed hind wing, a character otherwise exclusive to the extant lacewing family Nemopteridae, while its distal part of wings is not preserved in its holotype (Lu et al. 2017). *B. jiaxiaoae* sp. nov. possess several characters that are new for *Burmobabinskaia*. First, the hind wing of *B. jiaxiaoae* sp. nov. is narrow at the wing base but slightly broadened distad, while it is distinctly narrowed in the preserved part of *B. tenuis* . Second, the hind wing A1 is present in *B. tenuis* but lost in the new species. This condition is also present in the small‐sized babinskaiids genera *Electrobabinskaia* Lu et al. 2017, *Pseudobabinskaia* Makarkin et al. 2017, and *Paradoxoleon* Lu et al. 2022. Third, the female genitalia is first known for *Burmobabinskaia*, and the configuration is similar to that of *Electrobabinskaia burmana* Lu et al. 2017 by the coexistence of gonocoxites 8 and gonapophysis 8 (see in Hu et al. 2018). However, the new species lacks the projections on sternum 6 in comparison to *E. burmana*.

*Parababinskaia*, hitherto represented by three species and exhibiting the highest species diversity among babinskaiid genera, is further expanded by the new species described here. The hind wing of *P*. *weijie* sp. nov. possesses six presectoral crossveins, a trait uncommon in most known babinskaiids which typically have only 3–4. This condition was previously known only in the large‐sized, elongated‐winged genera *Calobabinskaia* Lu et al. 2021 and *Stenobabinskaia* Lu et al. 2021, indicating that the new species significantly expands the known morphological diversity of the family.

### 4.2 Evolutionary and Ecological Implications From Morphospace Disparity

Our morphospace analyses (Figure 8) reveal that Babinskaiidae from the Crato Formation and Kachin amber, while exhibiting little overlap, predominantly occupy distinct regions. The Kachin amber fauna exhibits markedly greater morphological disparity compared to the Crato Formation assemblage. This result is reflected in several distinct morphotypes: the slender, elongated wings with dense venation in *Calobabinskaia* and *Stenobabinskaia*; the sharply tapered wing tips in *Electrobabinskaia* and *Xiaobabinskaia* Lu et al. 2021; and the unique dilatation of all five metatarsomeres accompanied by elongated dilatation of all five metatarsomeres with curved claws in *Gigantobabinskaia* Makarkin and Staniczek, 2019 and *Electrobabinskaia*. The latter specialization on legs is thought to provide a selective advantage in an arboreal environment, facilitating better anchoring on leaf surfaces (Makarkin and Staniczek 2019).

The expanded morphospace occupied by the Kachin amber fauna, particularly along the PCoA1 axis of variation, appears to be strongly influenced by the inclusion of several large‐bodied genera, for example, *Gigantobabinskaia*, *Stenobabinskaia* and *Calobabinskaia*. The evolution of the significantly larger body size may have served as a key driver of morphological diversification. This pattern—where an increase in overall body size facilitates or correlates with greater morphological disparity—suggests that the Cretaceous ecosystems of Myanmar provided ecological opportunities that promoted this evolutionary expansion, as body size in insects is often related to dispersal ability, reproductive output, and ecological interactions such as predation or competition (Peters 1983; Honěk 1993; Woodward et al. 2005; Jenkins et al. 2007; Chown and Gaston 2010). Therefore, the disparity observed in the Myanmar assemblage may not merely reflect taxonomic diversity but could represent a more fundamental exploration of the available ecological morphospace within Babinskaiidae.

The ‘island rule’ predicts bidirectional body size shifts: gigantism in the smaller species and dwarfism in the larger species (Foster 1963, 1964, 1965; Van Valen 1973; Lawlor 1982), though this rule is still contentious (Lomolino 1985; McClain et al. 2006; Meiri et al. 2008). The mid‐Cretaceous Kachin babinskaiids provide a test case. The relatively large‐bodied taxa in Kachin amber exceed the typical sizes observed in Crato Formation babinskaiids (Figure 7), highlighting that body size expansion here does not reflect a violation of island rules but might rather reflect the ecological richness of the forested island habitat (Meiri et al. 2008; Warren et al. 2015). The extraordinarily rich Kachin amber biota reflects a highly productive and structurally complex tropical ecosystem, which might be driven by a warmer paleoclimate (Heimhofer et al. 2010; Metcalfe 2013; Tappert et al. 2013; Wang et al. 2014), with stratified vegetation and diverse microhabitats that likely provided a range of ecological opportunities (Warren et al. 2015; Yu et al. 2019). Such environmental heterogeneity may have relaxed ecological constraints and facilitated the expansion of morphological disparity within Babinskaiidae. The occurrence of large‐bodied taxa and specialized morphotypes thus suggests that these lineages experienced adaptive diversification under conditions that favored ecological experimentation and morphological innovation. This pattern underscores how insular but ecologically rich environments can serve as crucibles of morphological expansion, rather than restriction, during evolutionary history.

In contrast, the Crato Formation represents a lacustrine compression‐fossil deposit (Ribeiro et al. 2021), a setting that generally preserves larger insects than ambers due to taphonomic reasons. Therefore, the apparent absence of large individuals in the Crato Formation may be due to taphonomic bias. In addition, compared to ambers with fine three‐dimensional details, compression‐fossils generally lack fine morphological details of diagnostic characters such as legs and genitals, which might further contribute to the reduction of morphospace of Brazil babinskaiids. Additional material could clarify these issues. Overall, the expanded morphospace of the Kachin fauna likely reflects both genuine adaptive diversification and environmental complexity, highlighting the evolutionary flexibility of lacewings during the Late Cretaceous.

## Author Contributions

**Xiumei Lu:** conceptualization (lead), formal analysis (lead), funding acquisition (lead), methodology (lead), writing – original draft (lead), writing – review and editing (lead). **Yunlin Luo:** data curation (equal), software (equal), visualization (lead), writing – original draft (supporting). **De Zhuo:** resources (lead), writing – review and editing (equal). **Xingyue Liu:** conceptualization (lead), data curation (lead), funding acquisition (equal), resources (lead), supervision (lead), writing – original draft (equal), writing – review and editing (equal).

## Funding

This work was supported by the National Natural Science Foundation of China (Nos. 32370485, 31900348, 31672322) and the Royal Society Newton International Fellowship (Royal Society K.C. Wong Education Foundation) (NIF_R1_211688).

## Disclosure

*Specimen Collection Statement*: The authors attest that all legal and regulatory requirements, including export and import collection permits, have been followed for the collection of specimens from source populations at any international, national, regional, or other geographic level for all relevant field specimens collected as part of this study.

## Conflicts of Interest

The authors declare no conflicts of interest.

## Supporting information


**File S1:** ece373210‐sup‐0001‐DataS1.zip.

## Data Availability

We have included all data in both the article and its Supporting Information S1.
