# Supplementary material for: New Species of Babinskaiidae (Insecta: Neuroptera) From the Mid‐Cretaceous of Myanmar and the Morphological Divergence of the Family Across the Cretaceous
Source: Ecol Evol. 2026 Mar 9;16(3):e73210. doi: 10.1002/ece3.73210 (PMC12971284; doi:10.1002/ece3.73210)
Supplement: Supplementary file 1 — File S1: ece373210‐sup‐0001‐DataS1.zip. [file ECE3-16-e73210-s001.zip › ece373210-sup-0001-FileS1.doc]

Supplementary material for:

**New species of Babinskaiidae (Insecta: Neuroptera) from the mid-Cretaceous of Kachin amber and the morphological divergence of the family across the Cretaceous**

Xiumei Lu^1,2#^*, Yunlin Luo^3#^, De Zhuo^4^, Xingyue Liu^5^*

**Table S1. Checklist of Babinskaiidae (updated from Lu et al. 2022)**.

| **Family Babinskaiidae Martins-Neto & Vulcano, 1989** | | | |
| --- | --- | --- | --- |
| **No.** | **Species** | **Age** | **Locality** |
| 1 | *Baisonelia vitimica* Ponomarenko, 1992 | Lower Cretaceous, Barremian | Zaza Formation, Baissa, Siberia, Russia |
| 2 | *Babinskaia formosa* Martins-Neto & Vulcano, 1989a | Lower Cretaceous, Aptian | Crato Formation, Nova Olinda, Ceara, Brazil |
| 3 | *Babinskaia pulchra* Martins-Neto & Vulcano, 1989a | Lower Cretaceous, Aptian | Crato Formation, Nova Olinda, Ceara, Brazil |
| 4 | *Neliana maculata* (Martins-Neto & Vulcano, 1989b) | Lower Cretaceous, Aptian | Crato Formation, Nova Olinda, Ceara, Brazil |
| 5 | *Neliana impolluta* Martins-Neto, 1997 | Lower Cretaceous, Aptian | Crato Formation, Nova Olinda, Ceara, Brazil |
| 6 | *Parababinskaia elegans* Makarkin, Heads & Wedmann, 2017 | Lower Cretaceous, Aptian | Crato Formation, Nova Olinda, Ceara, Brazil |
| 7 | *Paraneliana sennlaubi* Jouault & Nel, 2021 | Lower Cretaceous, Aptian | Crato Formation, Nova Olinda, Ceara, Brazil |
| 8 | *Burmobabinskaia tenuis* Lu, Zhang & Liu, 2017 | mid-Cretaceous, Cenomanian | Tanai, Myitkyina, Kachin, Myanmar |
| 9 | *Calobabinskaia xiai* Lu, Wang & Liu, 2021 | mid-Cretaceous, Cenomanian | Tanai, Myitkyina, Kachin, Myanmar |
| 10 | *Electrobabinskaia burmana* Lu, Zhang & Liu, 2017 | mid-Cretaceous, Cenomanian | Tanai, Myitkyina, Kachin, Myanmar |
| 11 | *Gigantobabinskaia godunkoi* Makarkin & Staniczek, 2019 | mid-Cretaceous, Cenomanian | Tanai, Myitkyina, Kachin, Myanmar |
| 12 | *Stenobabinskaia punctata* Lu, Wang & Liu, 2021 | mid-Cretaceous, Cenomanian | Tanai, Myitkyina, Kachin, Myanmar |
| 13 | *Parababinskaia makarkini* Hu et al., 2018 | mid-Cretaceous, Cenomanian | Tanai, Myitkyina, Kachin, Myanmar |
| 14 | *Parababinskaia douteaui* Ngô-Muller et al., 2020 | mid-Cretaceous, Cenomanian | Tanai, Myitkyina, Kachin, Myanmar |
| 15 | *Pseudobabinskaia martinsnetoi* (Lu, Zhang & Liu, 2017) | mid-Cretaceous, Cenomanian | Tanai, Myitkyina, Kachin, Myanmar |
| 16 | *Pseudoneliana* *makarkini* Huang, Nel & Azar, 2019 | mid-Cretaceous, Cenomanian | Tanai, Myitkyina, Kachin, Myanmar |
| 17 | *Xiaobabinskaia lepidotricha* Lu, Wang & Liu, 2021 | mid-Cretaceous, Cenomanian | Tanai, Myitkyina, Kachin, Myanmar |
| 18 | *Paradoxoleon chenruii* Lu, Xu & Liu, 2022 | mid-Cretaceous, Cenomanian | Tanai, Myitkyina, Kachin, Myanmar |
| 19 | *Pseudelectrobabinskaia neli* (Jouault, 2022) | mid-Cretaceous, Cenomanian | Tanai, Myitkyina, Kachin, Myanmar |
| 20 | *Pseudelectrobabinskaia suae* Pu et al., 2025 | mid-Cretaceous, Cenomanian | Tanai, Myitkyina, Kachin, Myanmar |
| 21 | *Pseudelectrobabinskaia confuse* Pu et al., 2025 | mid-Cretaceous, Cenomanian | Tanai, Myitkyina, Kachin, Myanmar |
| 22 | *Microbabinskaia delicatula* Pu et al., 2025 | mid-Cretaceous, Cenomanian | Tanai, Myitkyina, Kachin, Myanmar |
| 23 | *Burmobabinskaia jiaxiaoae* sp. nov. | mid-Cretaceous, Cenomanian | Tanai, Myitkyina, Kachin, Myanmar |
| 24 | *Parababinskaia weijie* sp. nov. | mid-Cretaceous, Cenomanian | Tanai, Myitkyina, Kachin, Myanmar |
